# Fever of Unknown Origin Reveals a Missed Diagnosis of DiGeorge Syndrome in a 21-Year-Old Female

**DOI:** 10.7759/cureus.32355

**Published:** 2022-12-09

**Authors:** Nicole L Welch, Ashley Selman, Busara Songtanin, James A Tarbox

**Affiliations:** 1 Internal Medicine, Texas Tech University Health Sciences Center, Lubbock, USA; 2 Allergy and Immunology, Texas Tech University Health Sciences Center, Lubbock, USA

**Keywords:** adult diagnosis, chromosomal abnormalities, immunodeficiency, 22q11.2, digeorge syndrome

## Abstract

DiGeorge syndrome, caused by a microdeletion of the 22q11.2 region of chromosome 22, is a relatively rare condition. This syndrome can be difficult to recognize because a constellation of symptoms show different presentations. Most individuals diagnosed with this condition are identified in early childhood. With the emergence of new screening techniques, even fewer individuals with this syndrome are missed. Prior to these screening techniques, it was uncommon for patients to be diagnosed in adulthood. As a result, many internists, who focus only on the adult population, are unlikely to recognize and diagnose DiGeorge syndrome as the patient ages merely because it is not commonly diagnosed later in life. Early recognition and management are essential for the treatment of this condition. Here, we present the case of a 21-year-old woman diagnosed with DiGeorge syndrome as an adult.

## Introduction

DiGeorge syndrome, also known as 22q11.2 deletion syndrome, is a rare multisystem disorder but the most frequent microdeletion disorder with an incidence of approximately 1:4,000 [1,2]. This syndrome is typically diagnosed in young children less than two years old; however, a late diagnosis of DiGeorge syndrome (after 10 years of age) has been made, although the true incidence is unknown [3]. Newborn screening has been performed in the United States since 2008 through severe combined immunodeficiency (SCID) screening tests using quantitative PCR (qPCR) to recognize low levels of T-cell receptor excision circles (TRECs). This has increased the number of cases identified early in life. With the implementation of screening tests, even mild cases have been able to be identified early. However, some rare cases of DiGeorge syndrome are not diagnosed until adulthood.

DiGeorge syndrome is heterogeneous with a variable clinical picture. It can range from complete (severe) to partial (mild) forms. Most commonly, DiGeorge syndrome presents itself as a triad that includes immunodeficiency, congenital cardiac abnormalities, and hypoparathyroidism [4]. Being a primarily pediatric disease, most patients with DiGeorge syndrome enter adult medicine with a diagnosis. As a result, few internists have the experience needed to identify and diagnose this condition. If unrecognized, patients are at an increased risk for psychiatric disorders, recurrent infections, autoimmune disease, and other complications due to the medical condition [4]. This paper aims to increase the awareness of physicians regarding the missed diagnosis of DiGeorge syndrome in adult patients.

## Case presentation

A 21-year-old woman with a history of parietal lobe agenesis, global developmental delays, left-sided hearing loss, frequent otitis media as a child, cleft palate repair as an infant, chronic pain, and major depressive disorders was referred to an immunology clinic for the evaluation of daily fevers of unknown origin that had been ongoing for 15 months. She also had a history of frequent upper respiratory infections, sinusitis, and alternating constipation/ diarrhea and, in the past year, experienced multiple episodes of depression and anxiety.

The measurements of vital signs revealed a blood pressure of 95/68 mmHg, a heart rate of 107 beats per minute, and a temperature of 98.0°F. Laboratory tests showed a hemoglobin of 13.8 g/dL, white blood cell count of 5560/mcL, normal electrolytes except calcium of 8.56 mg/dL (8.8-10.5 g/dL), and magnesium of 2.0 mg/dL (1.6-2.4 mg/dL). Parathyroid hormone level was 17 pg/mL (15-65 pg/mL), and vitamin D level was 23 ng/mL (30-100 ng/mL). She had a documented history of hypocalcemia ranging from 7.5-8.7 g/dL six months prior to evaluation. The chest X-ray, urinalysis, and lupus anticoagulant and antinuclear antibodies were negative. For primary care, she was tested for several infections, such as EBV, CMV, Hepatitis A, respiratory viral panel by PCR, COVID PCR, *Streptococcus pneumonia*, influenza, and HIV, which were all negative. Gastroenterology ruled out Crohn’s disease and celiac disease; rheumatology excluded lupus and autoimmune vasculitides; neurology conducted magnetic resonance imaging of the temporomandibular joint to rule out any suspicion of dental infections and neurological causes from the differential for hyperthermia. An echocardiogram was performed, and it ruled out any structural cardiac defects and infective endocarditis.

An immunology workup was ordered and showed a C-reactive protein 0.9 mg/dL (0.0-0.5 mg/dL), a low CD4+ T cell count of 347/mcL (474-1,585/mcL), immunoglobulin A deficiency <5 mg/dL (70-400 mg/dL) with normal immunoglobulin G, and immunoglobulin M levels. Both the anti-thyroid peroxidase and antithyroglobulin antibodies were elevated at 242 IU/mL (0-34 IU/mL) and 424 IU/mL (0-115 IU/mL), respectively. Serology studies showed that the patient was protected against tetanus but not against *Streptococcus pneumonia* (titers showed protection against 7/23 strains; see Table 1). Chromosomal microarray analysis showed an abnormal fluorescent in situ hybridization result for the DiGeorge (TUPLE1) critical region.

**Table 1:** Results of the immunological workup performed on the patient with normal ranges for reference. ^a^ 7/23 titers above the cut-off value of 1.3 indicating a poor immune response to *S. pneumoniae* ^b^ 18/23 titers above the cut-off value of 1.3; 16/23 titers had a four-fold increase, indicating an adequate immune response to *S. pneumoniae.* ^c ^A cut-off value of 1.3 was used to determine serology response based on the existing literature [5]. ^d^ A normal lymphocyte mitogen response

Test	Result		Normal range
C-reactive protein	0.9 mg/dL		0.0-0.5 mg/dL
CD4+ T cell count	347/mcL		474-1,585 /mcL
Immunoglobulin A	<5 mg/dL		70-400 mg/dL
Immunoglobulin G	1602 mg/dL		700-1600 mg/dL
Immunoglobulin M	91 mg/dL		40-230 mg/dL
Anti-thyroid peroxidase	242 IU/mL		0-34 IU/mL
Antithyroglobulin antibodies	424 IU/mL		0-115 IU/mL
*S. pneumonia* Serology titers	Pre-vaccine titers Serotype 1 (1) <0.3 Serotype 2 (2) 1.0 Serotype 3 (3) <0.3 Serotype 4 (4) <0.3 Serotype 5 (5) 1.4 Serotype 8 (8) 0.4 Serotype 9 (9N) 0.4 Serotype12 (12F) <0.3 Serotype14 (14) 2.7 Serotype17 (17F) <0.3 Serotype19 (19F) 2.7 Serotype20 (20) 2.0 Serotype22 (22) <0.3 Serotype23 (23F) <0.3 Serotype26 (6B) <0.3 Serotype34 (10A) 0.4 Serotype43 (11A) <0.3 Serotype51 (7F) 1.3 Serotype54 (15B) 2.0 Serotype56 (18C) 0.5 Serotype57 (19A) 2.4 Serotype68 (9V) <0.3 Serotype70 (33F) 0.6 ^a^	Post-vaccine titers Serotype 1 (1) 5.4 Serotype 2 (2) 4.6 Serotype 3 (3) 0.6 Serotype 4 (4) 3.8 Serotype 5 (5) 8.5 Serotype 8 (8) 5.8 Serotype 9 (9N) 2.6 Serotype12 (12F) 1.2 Serotype14 (14) 13.7 Serotype17 (17F) 0.5 Serotype19 (19F) 10.2 Serotype20 (20) 4.4 Serotype22 (22) 3.1 Serotype23 (23F) <0.3 Serotype26 (6B) 2.4 Serotype34 (10A) 6.7 Serotype43 (11A) 1.4 Serotype51 (7F) 7.7 Serotype54 (15B) >81.0 Serotype56 (18C) 5.2 Serotype57 (19A) 23.0 Serotype68 (9V) 0.5 Serotype70 (33F) 4.3 ^b^	Antibody concentration greater than or equal to 1.3 µg/mL is generally considered long-term protection and used as a cutoff value to determine protective titers.
Lymphocyte Mitogen Response	Viab of Lymph at Day 0: 63.5%; Low Max Prolif of PWM as %CD45: 16.9%; Max Prolif of PWM as %CD3: 17.7%; Max Prolif of PWM as %CD19: 20.3%; Max Prolif of PHA as %CD45: 72.7%; Max Prolif of PHA as %CD3: 75.6%		Day 0: >75; %CD45: >4.5; %CD3: >3.5; %CD19: >3.9; %CD45: >49.9; %CD3: >58.5

This patient was subsequently diagnosed with DiGeorge syndrome and IgA deficiency. A pneumococcal vaccination series resulted in improved protection (16/23) against *S. pneumoniae*. The fever resolved without any diagnosis or source of infection. Though the patient had several courses of antibiotics over the 15 months prior to immunology referral, the daily fevers did not resolve. Presumably, the pneumococcal vaccine may have stimulated her immune system enough to allow the fevers to resolve. This may have been due to a chronic, low-grade sinusitis that went undiagnosed. After her symptoms were optimally managed, the patient is currently following up with her primary care provider, immunologist, and gastroenterologist every six months, ENT specialist every four to six months, and her psychiatrist as needed.

## Discussion

DiGeorge syndrome is a multisystem developmental disorder caused by a microdeletion of the 22q.11.2 chromosome, which is a *de novo* process in 90% of the cases, causing the improper formation of the third and fourth pharyngeal pouches during the 12th week of pregnancy [2,6]. It is usually diagnosed in young children and can present as a completely or partially absent thymus, cardiac anomalies, hypocalcemia, and facial abnormalities. Gastrointestinal problems, psychiatric disorders, and genitourinary conditions may also be present [4]. Typical physical manifestations are a cleft palate, low-set ears, down-slanting eyes, a short philtrum, a small mouth, and short stature [6]. A cardiovascular examination may reveal a murmur due to congenital cardiac anomalies [6-8]. Psychiatric manifestations include learning disabilities, developmental delays, or more serious psychiatric conditions such as schizophrenia, which can occur in up to 60% of patients [7,9]. The mortality rate was found to be 8%, with most dying within six months of birth from congenital heart disease; however, data for adult populations are limited, with some studies suggesting underestimated mortality [10,11].

DiGeorge syndrome is typically diagnosed in young children aged less than two years; since the implementation of newborn screening with TREC, this has become increasingly true [10,12]. By 2018, all states in the United States adopted this strategy. Despite the success of these screening programs, a few patients reach adulthood without the recognition of a disease as the testing may not reveal a diagnosis for their low TRECs, while others may not have had the opportunity to undergo screening [13]. One small study with 29 patients suggested the incidence of DiGeorge syndrome diagnosed after infancy was 31%, while another study showed only 4/12 (33%) case reports written on the delayed diagnosis of DiGeorge syndrome were in adults (>18 years old) [2,14].

In the case reported in this study, this patient had a missed diagnosis due to having partial DiGeorge syndrome. Her case was missed in infancy due to a mild initial presentation, a failure to recognize the constellation of symptoms as she aged, and the absence of TREC screening. This patient required a multitude of specialists to be involved in her care before other causes could be ruled out and DiGeorge syndrome could be concluded. The immunologist first saw the patient as an adult, after several other specialists had excluded other potential diagnoses. Given her overall history, combined with the surgical history of cleft palate repair, testing of the patient for DiGeorge syndrome was done at the age of 21.

Immunologic disorders occur in 75% of patients; the severity is related to the extent of thymic hypoplasia [7]. Complete absence of the thymus, a form of SCID, occurs in 1% of patients with chromosome 22q11 microdeletion syndrome and requires a thymic transplant. These patients have a poor prognosis and often die within a year. Partially athymic patients present with recurrent sinopulmonary infections but may be asymptomatic if a normal T-cell count is maintained. Although the patient in the case was diagnosed as if she had two individual diseases, DiGeorge syndrome is known to affect both humoral and cellular immunity. Therefore, the IgA deficiency is most likely a consequence of the effect of the 22q11.2 deletion syndrome on humoral immunity rather than a separate condition occurring simultaneously.

The long-term treatment involves the management of multiple potential complications by a multidisciplinary team of specialists, including cardiologists, endocrinologists, psychiatrists, immunologists, craniofacial surgeons, nutritionists, speech therapists, and other healthcare professionals. Screening includes auditory evaluation, growth charts, and psychiatric monitoring. Genetic counseling should be considered in patients old enough to reproduce; some patients require correction of palatal defects and close monitoring of immune/endocrine function, with a few patients even needing frequent vaccination boosters. Live vaccines should be considered on a case-by-case basis [15].

## Conclusions

The vast majority of DiGeorge syndrome cases are diagnosed at less than two years of age; the true incidence of adult diagnosis is unknown but is believed to be relatively rare. Thus, the education of clinicians of adult medicine is important to avoid a missed diagnosis. It is also important to perform appropriate screening and follow up with these patients. An evaluation for DiGeorge syndrome should be considered for any adult experiencing unexplained fevers and recurrent infections along with a history of intellectual disabilities, psychiatric conditions, hypocalcemia, cardiac symptoms, and/or cleft palate deformities. It is important to consider the overall medical history of the patient so as not to neglect something that might be critical. More studies are needed on this topic to determine the true incidence of DiGeorge syndrome in adulthood.
